# Examining Disparities in the Evaluation and Management of Cesarean Birth Pain Among Patients With and Without a Psychiatric Condition

**DOI:** 10.7759/cureus.78902

**Published:** 2025-02-12

**Authors:** Caitlyn B Rogers, Madeline J Pence, Julia Whitley, Anna Mattson, Sean M Lee, Jennifer Keller

**Affiliations:** 1 Pediatric Medicine, Children’s Hospital and Medical Center, Washington, DC, USA; 2 Obstetrics and Gynecology, University of Texas at Austin Dell Medical School, Austin, USA; 3 Obstetrics and Gynecology, Washington University School of Medicine, St. Louis, USA; 4 Obstetrics and Gynecology, Kaiser Permanente, Santa Clara, USA; 5 Obstetrics and Gynecology, George Washington University School of Medicine and Health Sciences, Washington, DC, USA

**Keywords:** cesarean section (cs), health disparities, mental health, pain management, postpartum pain

## Abstract

Background

Postoperative pain management is an important aspect of postpartum care following cesarean delivery (CD). This study aimed to determine whether differences exist in pain assessments and opioid administration after CD between patients with psychiatric illness and those without.

Methodology

This was a retrospective cohort study of 490 patients who underwent CD at an urban tertiary care center. Demographic and delivery data were collected by chart review. The primary outcomes were the number of pain assessments performed, average pain score, and amount of morphine milligram equivalents (MMEs) administered to patients with and without psychiatric illness.

Results

A total of 389 patients without a psychiatric diagnosis were compared to 101 patients with a psychiatric diagnosis. After adjusting for baseline characteristics in all models, psychiatric history had a significant effect on pain severity (β = 0.25; 95% confidence interval (CI) (0.00, 0.49); p = 0.046) and number of pain assessments (β = -2.41; 95% CI (-4.42, -0.41); p = 0.018), but not on MME administration (incidence rate ratio = 1.31; 95% CI (0.60, 2.92); p = 0.466). Patients with a psychiatric history reported more severe pain after CD and received fewer pain assessments. There was no significant difference in the amount of pain medication administered between groups.

Conclusions

In this study, patients with a psychiatric diagnosis received fewer pain assessments and reported more severe post-CD pain compared to those without. Despite this difference, both groups received similar amounts of pain medication, raising concern for bias and inadequate treatment of pain in patients with a psychiatric diagnosis.

## Introduction

Cesarean delivery (CD) is one of the most common surgeries performed in the United States, with 1.18 million CDs occurring in 2021 [1]. Pain management after delivery is critical for patient comfort, promoting maternal-infant bonding, and establishing lactation [2]. Perinatal mental illness is exceedingly common, with the prevalence of depression reaching 20% during pregnancy and the first three months postpartum [3], and untreated pain has been associated with postpartum depression [4]. A positive correlation between depression and increased perception of acute pain has been identified [5], and schizophrenia is associated with both increased sensitivity to acute pain [6] and elevated pain tolerance, with reduced physiologic response to noxious stimuli [7]. A better understanding of the relationship between acute pain, mental illness, and disparate care of patients with psychiatric diagnoses may be further elucidated by studying the post-CD period.

A review of the existing literature demonstrates that there is disparate treatment of pain in racial and ethnic minority patients across clinical settings. In the postpartum period, these patients have been found to receive less pain medication despite reporting more severe pain levels than non-minority patients [8,9]. A systematic review by Lee et al. found that racial and ethnic minorities were less likely to receive pain medication for acute pain in US emergency departments [10]. Similarly, bias against patients with mental illness is well-studied in many fields. This population has been shown to receive delayed treatment for breast cancer [11] and is less likely to receive a kidney transplant for chronic kidney disease [12] and the recommended pathology tests for diabetes [13] than patients without a psychiatric diagnosis. Whether similar disparities in addressing and treating postpartum pain exist among patients with a psychiatric diagnosis is not as well-studied. However, anxiety and depression have been associated with severe postpartum pain [14], and as such management of these patients after CD should be examined further.

Several studies evaluating post-CD pain severity and management in patients with a psychiatric diagnosis have found that patients with a psychiatric history report more severe postpartum pain [15,16], while others have found no difference in postpartum pain severity in this population [14]. Studies of opiate administration in patients with a psychiatric history are similarly conflicting, with Emerson et al. [17] demonstrating higher postpartum opiate use among these patients, while Ozturk et al. [15] found that a significant level of prenatal anxiety was not associated with an increased analgesic requirement despite an association with more severe post-CD pain. Notably, these studies differed in the psychiatric diagnoses included and the length of time during which analgesic medication administration was studied.

Our study has two objectives. The first is to determine whether differences exist in the frequency of pain evaluations performed after CD among patients with versus without a psychiatric diagnosis. We hypothesize that patients with a psychiatric diagnosis receive fewer pain assessments after CD. Our second objective is to determine whether differences exist in opioid administration after CD between patients with versus without a psychiatric diagnosis. We hypothesize that fewer opioids (as measured by morphine milligram equivalents (MMEs)) are administered to patients with a psychiatric diagnosis during admission and on hospital discharge.

## Materials and methods

We performed a retrospective cohort study of patients who underwent CD at George Washington (GW) University Hospital, an urban tertiary care center located in Washington, DC, from January 1, 2019, to December 31, 2019. Approval for this study was obtained from the GW University Hospital institutional review board as an exempt study (approval number: #NCR202470). A confidential list of all patients undergoing cesarean birth during the study period was obtained from a Clinical Systems Analyst at GW Hospital, which was used to obtain electronic medical record (EMR) data. Three study team members (CR, AM, MP) performed manual chart review and data extraction. All study authors were primarily affiliated with GW Hospital at the time of data collection.

Patients ≥18 years of age were included. Patients with a duplicate chart were excluded. Sociodemographic data were extracted, including patient age, self-reported race, insurance status, marital status, and primary language spoken. Clinical data recorded include maternal body mass index (BMI) at delivery, past medical history, psychiatric history, antidepressant use, antipsychotic use, substance use, parity, prior CD, history of prior abdominal surgery, CD indication, gestational age in weeks, multiple gestation, admission hemoglobin, pregnancy or delivery complications. Patients were considered to have a psychiatric diagnosis if they had one of the following diagnoses documented in their hospital admission note or on the problem list in the EMR: schizophrenia, schizoaffective disorder, bipolar disorder, depression, anxiety, post-traumatic stress disorder, and/or obsessive-compulsive disorder. Postoperative data collected included length of hospital stay, neonate weight, postoperative hemoglobin, number of pain assessments performed, mean pain score, and MMEs administered.

The number of pain assessments performed from the end of CD to the time of hospital discharge was totaled for each patient. A pain assessment was included if the nursing staff performed and charted either a numeric pain scale ranging from 0 to 10 or the Faces Pain Scale [18]. The mean pain score was calculated as the average of the pain scores obtained from these assessments. Postpartum nursing staff administered analgesic medication according to a standard post-CD order set that allowed nurses to offer opioids based on the pain score reported by the patient. The medical administration record was utilized to calculate the amount of opioid pain medication administered. Opioid medications administered to the included patients at our institution were oxycodone and hydromorphone. An MME calculator was utilized to standardize the amount of pain medication each patient received [19]. A non-opioid pain management regimen was also ordered for all patients after CD at GW according to a standard protocol consisting of (1) scheduled oral acetaminophen 1 g every eight hours and (2) scheduled intravenous ketorolac 30 mg every six hours which was transitioned to scheduled ibuprofen 600 mg every eight hours for the duration of the hospital stay.

To compare demographic and baseline characteristics between the group with and without a psychiatric diagnosis, we employed chi-squared tests of independence for categorical variables and two-sided t-tests assuming unequal variances for continuous variables. We then fitted linear models to data representing each of the following outcomes: pain severity, pain assessment, and morphine equivalents. We fitted each model as a function of the presence of psychiatric diagnosis. In all three linear models, we adjusted for demographic and baseline characteristics for which p-value <0.20 in univariate analyses by including them as covariates. For pain severity and pain assessment, we fitted robust linear models to adjust for extreme values. Because morphine equivalents were right-skewed and comprised zeros, we fitted a generalized linear model with a negative binomial error distribution. We adjusted for pain severity in the generalized linear model by including an interaction term between the presence of psychiatric diagnosis and pain severity. The interaction term allowed us to test whether the effect of a psychiatric diagnosis on morphine equivalent depends on pain severity. For each of the three models, we performed hypothesis tests for each predictor variable with an alpha level of 5%. Statistical analyses were performed using the R Core Team [20].

## Results

There were a total of 2,111 live births during the study period, 490 of which were CDs. Indications for CD included non-reassuring fetal heart tracing (n = 142), failure to progress (n = 156), malpresentation (n = 79), prior CD (n = 179), multiple gestation (n = 20), placenta previa (n = 11), prior uterine surgery (n = 9), elective CD (n = 33), and “other,” which included suspected placental abruption (n = 2) and early labor with an abdominal cerclage (n = 1).

Approximately one-fifth (n = 101, 20.6%) of the 490 patients in the study had a psychiatric diagnosis documented in the EMR. Demographic characteristics of patients with and without psychiatric history are shown in Table 1. The psychiatric diagnoses included and the number of patients with each is shown in Table 2. Table 3 demonstrates the mean and median values for pain score, number of pain assessments, and MMEs administered to patients with versus without a psychiatric diagnosis.

**Table 1:** Demographic characteristics of included patients. BMI: body mass index; CD: cesarean delivery

	Psychiatric diagnosis		P-value
	No (N = 389)	Yes (N = 101)	
Age			
Mean (SD)	32.1 (6.05)	32.0 (5.87)	0.802
Median (minimum, maximum)	33.0 (17.0, 50.0)	33.0 (19.0, 44.0)	
BMI			
Mean (SD)	33.2 (8.20)	33.3 (8.79)	0.856
Median (minimum, maximum)	31.8 (18.2, 87.5)	31.3 (19.4, 63.3)	
Race			
Black, N (%)	189 (48.6%)	54 (53.5%)	0.0144
White, N (%)	108 (27.8%)	37 (36.6%)	
Asian, N (%)	25 (6.4%)	1 (1.0%)	
Hispanic/Latino, N (%)	8 (2.1%)	3 (3.0%)	
Other, N (%)	59 (15.2%)	6 (5.9%)	
Operative time			
Mean (SD)	101 (37.8)	98.8 (26.4)	0.561
Median (minimum, maximum)	94.0 (0, 501)	93.0 (58.0, 193)	
Past medical history			
None, N (%)	135 (34.7%)	40 (39.6%)	0.424
One or more conditions, N (%)	254 (65.3%)	61 (60.4%)	
Delivery complications			
None, N (%)	99 (25.4%)	28 (27.7%)	0.736
At least one complication, N (%)	290 (74.6%)	73 (72.3%)	
Pregnancy complications			
None, N (%)	128 (32.9%)	31 (30.7%)	0.761
At least one, N (%)	261 (67.1%)	70 (69.3%)	
Estimated blood loss			
Mean (SD)	833 (408)	813 (394)	0.662
Median (minimum, maximum)	740 (80.0, 2,830)	720 (220, 2,420)	
Neonate weight			
Mean (SD)	3,200 (756)	3,040 (796)	0.0623
Median (minimum, maximum)	3,250 (410, 4,820)	3,190 (475, 4,690)	
Gestational age			
Mean (SD)	38.1 (2.85)	38.0 (3.67)	0.66
Median (minimum, maximum)	39.0 (23.0, 42.0)	39.0 (24.0, 42.0)	
Parity			
Nulliparous, N (%)	177 (45.5%)	52 (51.5%)	0.336
Multiparous, N (%)	212 (54.5%)	49 (48.5%)	
Insurance type			
Public, N (%)	178 (45.8%)	49 (48.5%)	0.468
Private, N (%)	208 (53.5%)	50 (49.5%)	
Other, N (%)	3 (0.8%)	2 (2.0%)	
Relationship status			
Single, N (%)	160 (41.1%)	56 (55.4%)	0.0357
Married, N (%)	219 (56.3%)	43 (42.6%)	
Unknown, N (%)	10 (2.6%)	2 (2.0%)	
Substance use			
None, N (%)	370 (95.1%)	81 (80.2%)	<0.001
At least one, N (%)	19 (4.9%)	20 (19.8%)	
Prior abdominal surgery			
None, N (%)	193 (49.6%)	61 (60.4%)	0.0687
At least one, N (%)	196 (50.4%)	40 (39.6%)	
Previous CD			
None, N (%)	214 (55.0%)	66 (65.3%)	0.0789
At least one, N (%)	175 (45.0%)	35 (34.7%)	
Change in hemoglobin			
Mean (SD)	-1.84 (1.56)	-1.75 (1.49)	0.597
Median (minimum, maximum)	-1.80 (-10.3, 10.3)	-1.80 (-5.40, 8.82)	
Hospital length of stay			
Mean (SD)	3.10 (0.753)	3.18 (0.792)	0.376
Median (minimum, maximum)	3.00 (1.00, 8.00)	3.00 (2.00, 9.00)	
Gestation			
Singleton, N (%)	364 (93.6%)	96 (95.0%)	0.179
Twin, N (%)	23 (5.9%)	3 (3.0%)	
Unknown, N (%)	2 (0.5%)	2 (2.0%)	

**Table 2:** Psychiatric diagnoses. *: Not mutually exclusive as some individuals can fall under multiple diagnoses.

Psychiatric diagnosis (N = 490)	N (%)
0 (none)	389 (79.4%)
1 (at least 1)	101 (20.6%)
Depression	58 (11.8%)
Anxiety	53 (10.8%)
Bipolar disorder	9 (1.8%)
Obsessive-compulsive disorder	1 (0.2%)
Schizophrenia	1 (0.2%)
Schizoaffective disorder	1 (0.2%)
Other	9 (1.8%)

**Table 3:** Pain severity, pain assessments, and MMEs between groups. MME: morphine milligram equivalent

	Psychiatric diagnosis		
	No (N = 389)	Yes (N = 101)	Total (N = 490)
Pain severity			
Mean (SD)	2.18 (1.06)	2.53 (1.36)	2.25 (1.14)
Median (minimum, maximum)	2.04 (0, 7.79)	2.33 (0.300, 6.93)	2.09 (0, 7.79)
Number of pain assessments			
Mean (SD)	23.3 (12.0)	21.9 (11.2)	23.0 (11.8)
Median (minimum, maximum)	21.0 (3.00, 98.0)	20.0 (5.00, 64.0)	21.0 (3.00, 98.0)
MME administered			
Mean (SD)	58.0 (61.7)	68.8 (69.4)	60.2 (63.4)
Median (minimum, maximum)	37.5 (0, 360)	45.0 (0, 263)	45.0 (0, 360)

**Figure 1:**
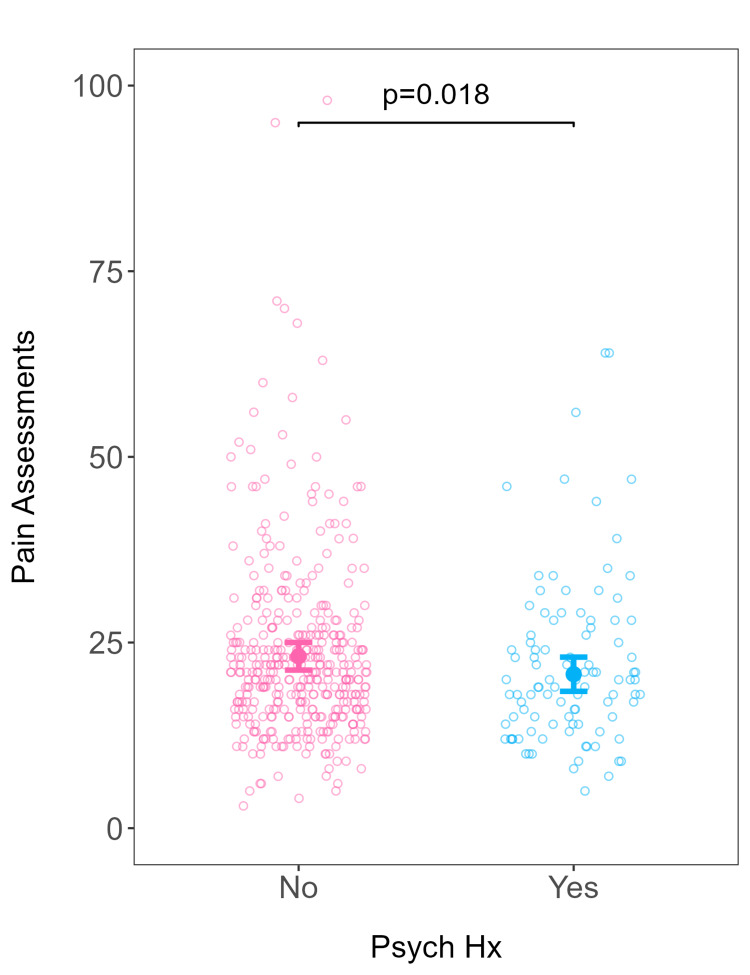
Number of pain assessments as a function of psychiatric diagnosis. Predicted number of pain assessments (filled circles) and 95% confidence intervals from the robust linear model fitted as a function of psychiatric history, adjusting for relevant covariates. All covariates held constant at their respective baseline values. Open circles represent actual data from individual patients. Psych Hx: psychiatric history

**Figure 2:**
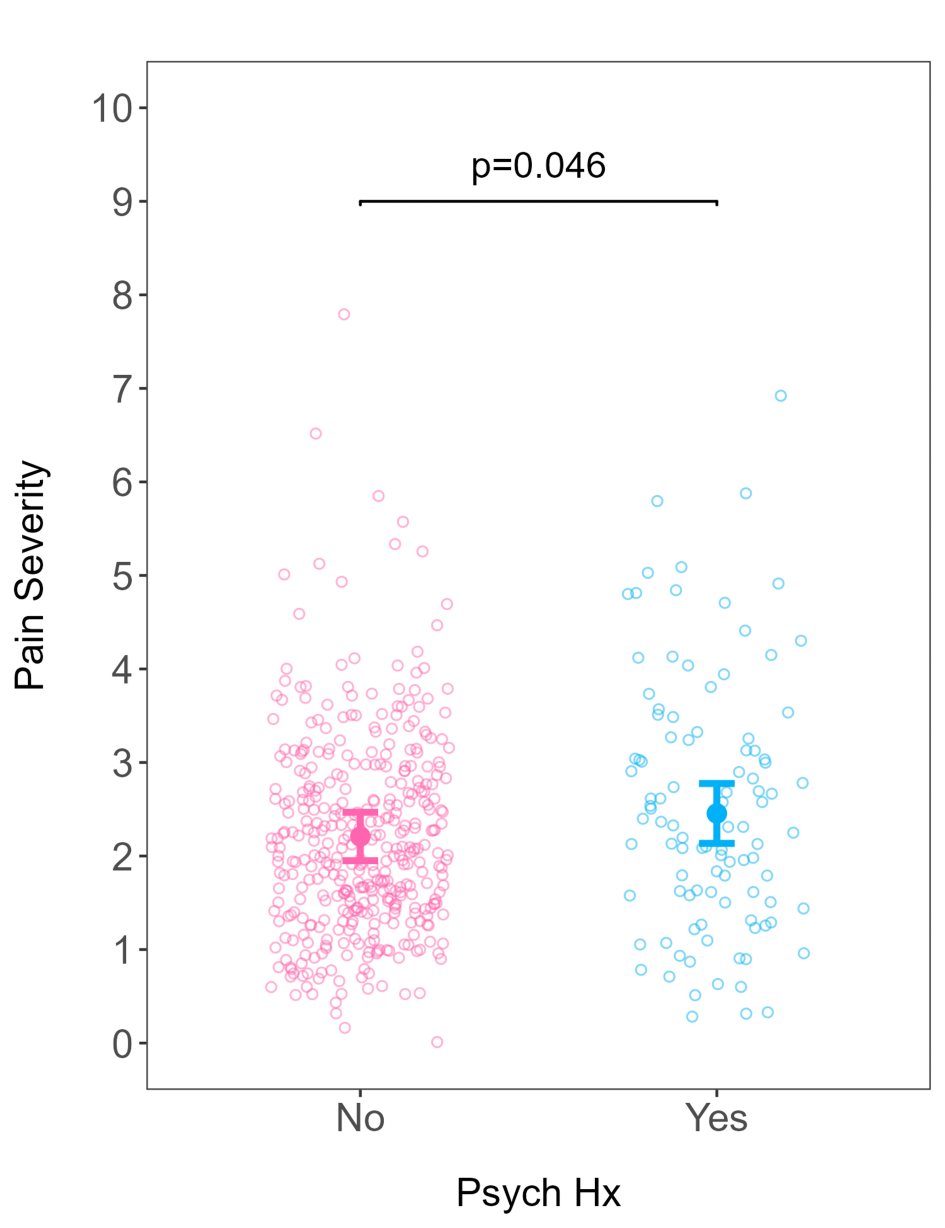
Pain severity as a function of psychiatric diagnosis. Predicted pain severity (filled circles) and 95% confidence intervals from the robust linear model fitted as a function of psychiatric history, adjusting for relevant covariates. All covariates held constant at their respective baseline values. Open circles represent actual data from individual patients. Psych Hx: psychiatric history

**Figure 3:**
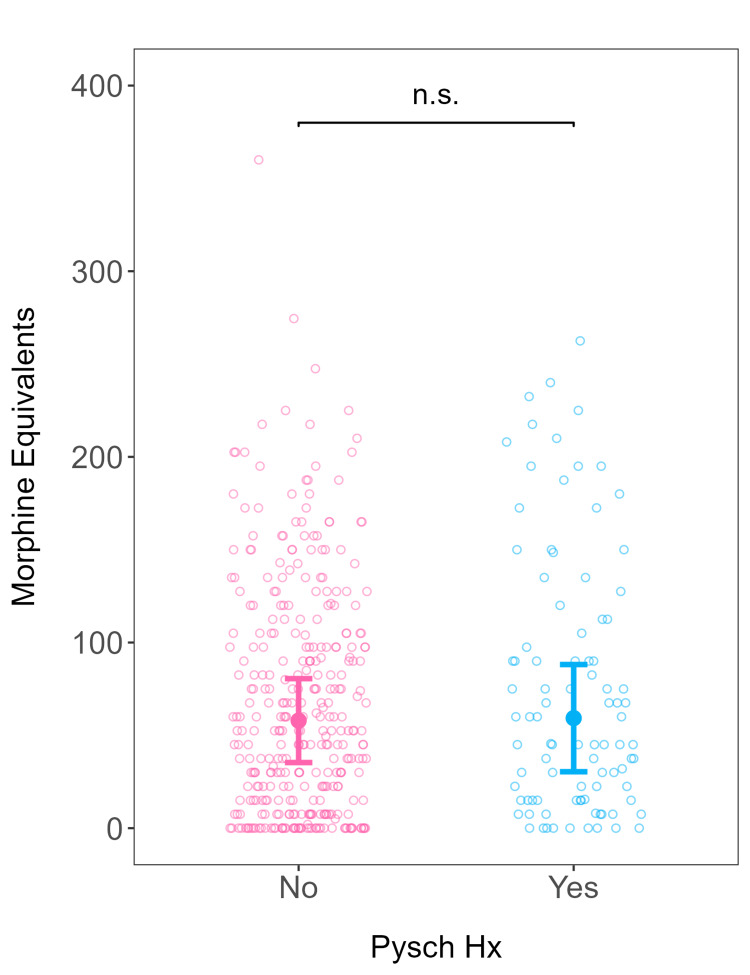
MMEs administered as a function of psychiatric diagnosis. Predicted morphine equivalents (filled circles) and 95% confidence intervals from the generalized linear model fitted as a function of psychiatric history, adjusting for relevant covariates. All covariates held constant at their respective baseline values. Open circles represent actual data from individual patients. Psych Hx: psychiatric history; MME: morphine milligram equivalent

When adjusting for covariates among patients, more pain assessments were conducted on patients without a psychiatric diagnosis compared to those with a psychiatric diagnosis (β = -2.41; 95% confidence interval (CI) (-4.42, -0.41); p = 0.018) (Table 4; Figure 1). Similarly, nulliparous patients received more pain assessments than multiparous patients (Table 4). In terms of pain severity, patients with a psychiatric diagnosis had higher predicted pain severity than patients without a psychiatric diagnosis (β = 0.25; 95% CI (0.00, 0.49); p = 0.046) (Table 5; Figure 2). There was no difference between the number of MMEs administered to patients with and without a psychiatric diagnosis when adjusted for pain severity (incidence rate ratio = 1.31; 95% CI (0.60, 2.92); p = 0.466) (Table 6; Figure 3).

**Table 4:** Pain assessment model summary. Coefficient estimates and 95% CIs, SEs, and t and p-values from the robust linear model fitted to data representing pain assessments as a function of psychiatric diagnosis, adjusting for relevant covariates. CI: confidence interval; SE: standard error; CD: cesarean delivery

Coefficient	Estimate	CI	SE	t-value	P-value
Intercept	26.41	22.63–30.18	1.92	13.74	<0.001
Psychiatric diagnosis: yes	-2.41	-4.42–-0.41	1.02	-2.36	0.018
Race: Black (reference)	–	–	–	–	–
Race: White	-0.88	-3.22–1.45	1.19	-0.75	0.457
Race: Asian	-1.67	-5.58–2.24	1.99	-0.84	0.402
Race: Hispanic/Latino	-0.29	-5.70–5.11	2.75	-0.11	0.915
Race: Other	-0.04	-2.76–2.69	1.39	-0.03	0.978
Neonate weight	-0.00	-0.00–0.00	0.00	-1.41	0.158
Married: yes	-0.81	-3.00–1.38	1.12	-0.73	0.468
Married: unknown	0.40	-4.79–5.59	2.64	0.15	0.879
Substance use: yes	2.10	-0.98–5.18	1.57	1.34	0.182
Previous abdominal surgery: yes	-2.72	-6.15–0.71	1.74	-1.56	0.120
Previous CD: yes	-0.86	-4.32–2.60	1.77	-0.49	0.626
Multiple gestation: yes	0.87	-2.79–4.53	1.86	0.46	0.642
Multiple gestation: unknown	3.07	-5.65–11.79	4.43	0.69	0.490

**Table 5:** Pain severity model summary. Coefficient estimates and 95% CIs, SEs, and t and p-values from the robust linear model fitted to data representing pain severity as a function of psychiatric diagnosis, adjusting for relevant covariates. CI: confidence interval; SE: standard error; CS: cesarean section

Coefficient	Estimate	CI	SE	t-value	P-value
Intercept	2.57	2.11–3.02	0.23	11.08	<0.001
Psychiatric diagnosis: yes	0.25	0.00–0.49	0.12	2.00	0.046
Race: Black (reference)	–	–	–	–	–
Race: White	-0.35	-0.63–-0.07	0.14	-2.47	0.014
Race: Asian	-0.26	-0.73–0.21	0.24	-1.09	0.278
Race: Hispanic/Latino	-0.09	-0.74–0.56	0.33	-0.28	0.781
Race: Other	-0.08	-0.41–0.25	0.17	-0.47	0.638
Neonate weight	-0.00	-0.00–0.00	0.00	-0.28	0.781
Married: yes	-0.30	-0.56–-0.04	0.13	-2.23	0.026
Married: unknown	-0.13	-0.76–0.49	0.32	-0.41	0.682
Substance use: yes	-0.10	-0.47–0.27	0.19	-0.52	0.602
Previous abdominal surgery: yes	-0.07	-0.48–0.34	0.21	-0.32	0.747
Previous CS: yes	-0.03	-0.45–0.39	0.21	-0.14	0.890
Multiple gestation: yes	-0.44	-0.88–0.01	0.22	-1.94	0.053
Multiple gestation: unknown	0.92	-0.13–1.97	0.53	1.71	0.087

**Table 6:** MME model summary Coefficient estimates, SEs, z values, IRRs and 95% CIs, and p-values from generalized linear model fitted to data representing morphine equivalents as a function of psychiatric history, adjusting for relevant covariates. SE: standard error; IRR: incidence rate ratio; CI: confidence interval; CS: cesarean section; MME: morphine milligram equivalent

Coefficient	Estimate	SE	z	IRR	CI	P-value
Intercept	3.10	0.38	8.09	22.25	10.37–49.32	<0.001
Psychiatric diagnosis: yes	0.27	0.37	0.72	1.31	0.60–2.92	0.466
Pain severity	0.49	0.07	6.53	1.63	1.39–1.91	<0.001
Psychiatric diagnosis × pain severity	-0.11	0.13	-0.82	0.90	0.67–1.21	0.413
Race: Black (reference)	–	–	–	–	–	–
Race: White	-0.33	0.21	-1.59	0.72	0.47–1.09	0.111
Race: Asian	-1.10	0.35	-3.14	0.33	0.17–0.70	0.002
Race: Hispanic/Latino	-1.80	0.49	-3.70	0.16	0.07–0.51	<0.001
Race: Other	-0.24	0.24	-1.00	0.79	0.48–1.30	0.318
Neonate weight	-0.00	0.00	-0.23	1.00	1.00–1.00	0.821
Married: yes	-0.08	0.20	-0.39	0.93	0.62–1.38	0.700
Married: unknown	-0.04	0.46	-0.10	0.96	0.42–2.71	0.927
Substance use: yes	0.05	0.27	0.19	1.05	0.64–1.84	0.850
Previous abdominal surgery: yes	-0.39	0.31	-1.28	0.68	0.38–1.32	0.200
Previous CS: yes	0.54	0.31	1.74	1.71	0.88–3.07	0.081
Multiple gestation: yes	0.20	0.33	0.62	1.22	0.67–2.45	0.535
Multiple gestation: unknown	-0.38	0.78	-0.49	0.68	0.18–5.18	0.626

## Discussion

Key finding 1: Patients with a psychiatric diagnosis received fewer pain assessments than patients without

In our retrospective cohort of women after cesarean birth, patients with a psychiatric history had fewer pain assessments compared to patients without a psychiatric history. This clinically significant difference persisted even when covariates were adjusted for, suggesting inequalities specific to pain management for patients with mental illness. These findings highlight prior work demonstrating the discrimination that patients with mental illness face in the clinical setting. In a systematic review of the impact of stigma and discrimination on nursing care for patients with mental illness, the most common experience shared by patients was under-treatment of physical symptoms. Additionally, nurses in this study expressed an attitude of futility in treating these patients and felt it was more challenging to build rapport with them [21]. One study specifically examining provider judgments of patients in pain also emphasized the tendency of providers to attribute physical symptoms of pain to psychological causes, which has been shown to affect both diagnostic and treatment decisions [22]. While it has been shown that discrimination against patients with mental health disorders occurs both at the individual level and the structural level within the field of healthcare, our study helps elucidate how this occurs from a pain management perspective during the immediate postpartum period.

Key finding 2: Nulliparous patients had more pain assessments than multiparous patients

This study also found that nulliparous patients had more pain assessments compared to those who were multiparous. Prior studies have demonstrated differences in opioid prescribing based on parity; however, this is the first study to our knowledge to report on differences in pain assessments based on parity following CD. In a multicenter study of opioid prescribing trends, primiparous women were more likely to receive opioids than multiparous women [23]. This may be explained by the notion that primiparous patients experience more fear, are more sensitive to in-labor pain, and are more likely to request pain relief. However, despite these findings, we believe that parity should not be a crucial determining factor when prescribing opioids to postpartum patients.

Key finding 3: When assessed, patients with a psychiatric diagnosis reported more severe pain than those without

In this study, patients with a psychiatric history reported more severe pain, when assessed, compared to those without a psychiatric history. Our finding aligns with prior work that demonstrated that patients with preexisting anxiety and/or depression had greater pain scores following CD [15,16]. Additionally, a prior study examining postoperative pain following laparoscopic tubal ligation found that several psychometric variables, including the State-Trait Anxiety Inventory and the Hospital Anxiety and Depression Scale, correlated with more severe postoperative pain [24]. However, a recent large cohort study of postpartum patients who underwent CD demonstrated no difference in pain scores between patients with and without a psychiatric diagnosis [14]. The conflicting literature demonstrates that the relationship between psychiatric disorders and pain severity is complex and likely unique to various conditions. An alternative interpretation of our study’s results is that patients with a psychiatric history may have reported more severe pain as a result of receiving fewer pain assessments and perhaps fewer doses of non-narcotic analgesics (which were not assessed in this study), resulting in more severe pain.

Key finding 4: When adjusting for pain severity, there was no significant difference in the amounts of opioids administered to both groups

In our study, patients with a psychiatric diagnosis received more opioids after CD than patients without a psychiatric diagnosis. However, after adjusting for pain severity, this difference was not significant. Studies with designs similar to ours have also demonstrated higher opioid use after CD in patients with a psychiatric history. Poehlmann et al. found that patients with anxiety had significantly higher MME use than patients without, and Emerson et al. found that a history of psychiatric comorbidity was associated with higher outpatient opioid use, although this finding was not significant [16,17]. It is possible that our study’s findings were non-significant because data was collected up to hospital discharge rather than 24 hours after CD. Interestingly, Ozturk et al. found that significant prenatal anxiety was associated with more severe post-CD pain but not with an increased analgesic requirement, which may explain why patients with a psychiatric history in our study, 10.8% of whom had a diagnosis of anxiety, did not receive significantly more opioids [15]. A final possibility is that the reliance on standard order sets for a given pain severity score at our institution, taken together with fewer overall pain assessments for patients with a psychiatric history, resulted in a non-significant difference in the amount of opioids given between groups.

Study strengths

One of the strengths of this study is its inclusion of a large, diverse cohort of patients who underwent CD at an urban hospital. Additionally, while similar studies have focused on patients with depression and/or anxiety [16], the psychiatric diagnoses included in our study were broad, particularly with the inclusion of schizophrenia, which has been previously shown to impact pain severity but is less well-studied than depression and anxiety [6]. Several potential confounders, including pregnancy and delivery complications, gestational age, parity, total CD operative time, and substance use, were also included and adjusted for through linear regression.

Limitations

This study’s retrospective design was performed with patients who delivered at a single center, which may limit its generalizability. Another limitation is that only patients with a documented psychiatric diagnosis in the EMR were included in the study group. This required the provider who wrote the labor and delivery admission note to explicitly elicit (and the patient to disclose) such a history or that the diagnosis was previously documented in the patient’s chart. The sensitive nature of many psychiatric diagnoses likely precluded the inclusion of all patients with a psychiatric diagnosis from being identified as such, which may have influenced the results. Further, although we did not identify any patients with opioid use disorder or current opioid use at the time of CD via chart review, it is possible that some patients included in the study were opioid-tolerant at the time of CD, had higher opioid requirements for pain control, and may have required more MMEs compared to opioid-naïve patients.

Future directions

In the immediate post-CD period, assessing and treating pain adequately is important. A significant association has been found between the severity of pain in the immediate postoperative period and the risk of developing persistent postpartum pain and depression [25]. Patients with a preexisting psychiatric diagnosis have been shown to have more severe postoperative pain [16]. In our study, such patients had fewer pain assessments. They did not receive significantly more pain medication despite reporting more severe pain, suggesting a potential bias by healthcare providers toward patients with psychiatric diagnoses. At the same time, patients with a psychiatric history have been shown to have higher rates of chronic opioid use after CD [17,25], making pain control in the postpartum period particularly imperative for these patients to prevent opioid dependence. To do this, existing strategies such as utilizing standardized practices for assessing pain with a consistent frequency and administering medication according to an order set may be used. Implementation and study of alternative measures to ensure equitable treatment of post-CD pain is an area for potential further study.

## Conclusions

In summary, in this retrospective cohort of patients birthing at a tertiary academic medical center in Washington, DC, patients with a psychiatric history received fewer pain assessments in the immediate postpartum period and reported more severe pain in comparison to patients without a psychiatric history. These differences were not explained by clinical factors associated with postoperative pain control, suggesting that differential treatment based on preexisting mental illness may be contributing to inequities in postpartum care.
